# Epithelioid sarcoma of the upper limb with nine years of evolution^☆^^☆☆^

**DOI:** 10.1016/j.abd.2020.06.007

**Published:** 2020-11-19

**Authors:** Maraya de Jesus Semblano Bittencourt, Caren dos Santos Lima, Aline de Lima Dias, Camilla Côrrea Neri

**Affiliations:** Department of Dermatology, Centro Universitário do Estado do Pará, Belém, PA, Brazil

*Dear Editor,*

Epithelioid sarcoma (ES) is a rare histopathological subtype of soft tissue sarcoma, accounting for less than 1% of all adult soft tissue sarcomas.^1^ It mainly affects the limbs of young patients and involves dermis, subcutaneous tissue, or deeper soft tissues. The etiology remains unknown, with a high percentage of lymph node and lung metastasis.^2^ The authors report a case of a 49-year-old male patient, who presented multiple painful ulcers on the left upper limb for nine years, which increased in number and extension and did not heal. The condition started with a left wrist injury after blunt trauma with a hammer. Upon dermatological examination, the authors observed the presence of multiple ulcers with bloody, elevated, infiltrated edges, some with a purulent floor, others with a granular and bloody floor, grouped, of varying sizes, affecting the entire extension of the left upper correct to CC BY limb (Figure 1, Figure 2). There were also nodules, one of them soft and erythematous-violaceous, and others with exulcerated surfaces, in a linear distribution on the left shoulder. He presented weight loss of 10 kg in the last year. The patient also had severe and continuous pain, requiring frequent use of analgesics and anti-inflammatory drugs. He had already undergone several oral, parenteral, and local antibiotic therapies, without improvement. The initial hypotheses were mycobacteriosis and deep mycoses. Chest radiography showed linear dense areas in the pulmonary bases and a small nodular image projected in the left lung field measuring approximately 0.3 cm. Histopathological examination showed an infiltrative neoplasm consisting of epithelioid and spindle cells with eosinophilic cytoplasm and irregular nuclei with evident nucleoli, fibrosis of the adjacent tissue, and focal necrosis (Fig. 3). The immunohistochemical examination showed expression for cytokeratins AE1/AE3, CD34, and complete loss of INI-1 expression. These findings are consistent with the diagnosis of ES. The ES initially presents as painless, localized, slowly growing nodules, which evolve to chronic ulcers that do not heal, with raised margins, usually in the distal limbs of young adults, although there are reports of cases affecting children and the elderly. Despite its slow growth, it can be an extremely aggressive tumor with a clinical course characterized by high rates of local recurrence and metastatic potential, especially for lymph nodes and lungs.^3^, ^4^ Histopathologically, it presents a nodular arrangement of epithelioid neoplastic cells with central degeneration and necrosis. Vascular invasion is rare. Neoplastic cells are oval or polygonal and large, similar to rhabdomyosarcoma. The spindle cells resemble fibrosarcoma or malignant fibrohistiocytoma.^2^ In immunohistochemical analysis, ES characteristically shows reactivity for epithelial markers, as well as for mesenchymal markers. It is consistently positive for cytokeratin, EMA, and vimentin expression. There is a positive reaction with CD34 in almost 50% of cases. INI-1 is deficient in approximately 90% of cases.^1^, ^4^ Complete surgical resection is the basis of curative therapy for ES. Some authors strongly recommend radical local excision, with amputation, as the first operative procedure.^5^ The authors report a rare case of upper limb ES, with long evolution before the diagnosis; attention is drawn to the important role of the dermatologist in the clinical recognition of this neoplasm.

**Figure 1:**
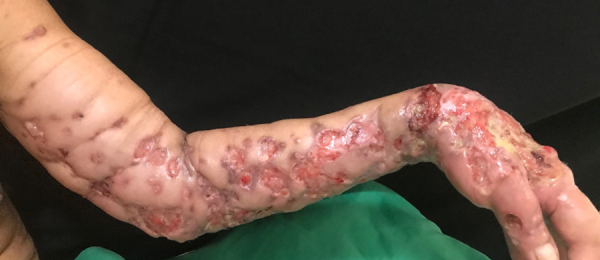
Multiple ulcers with raised and infiltrated edges, some with a purulent floor and others with a granular and bloody floor.

**Figure 2:**
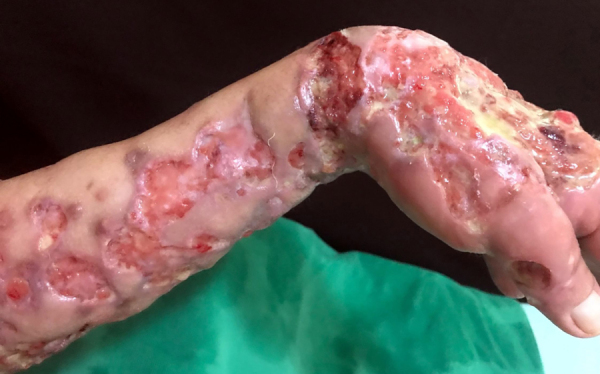
Detail of multiple ulcers with raised and infiltrated edges.

**Fig. 3:**
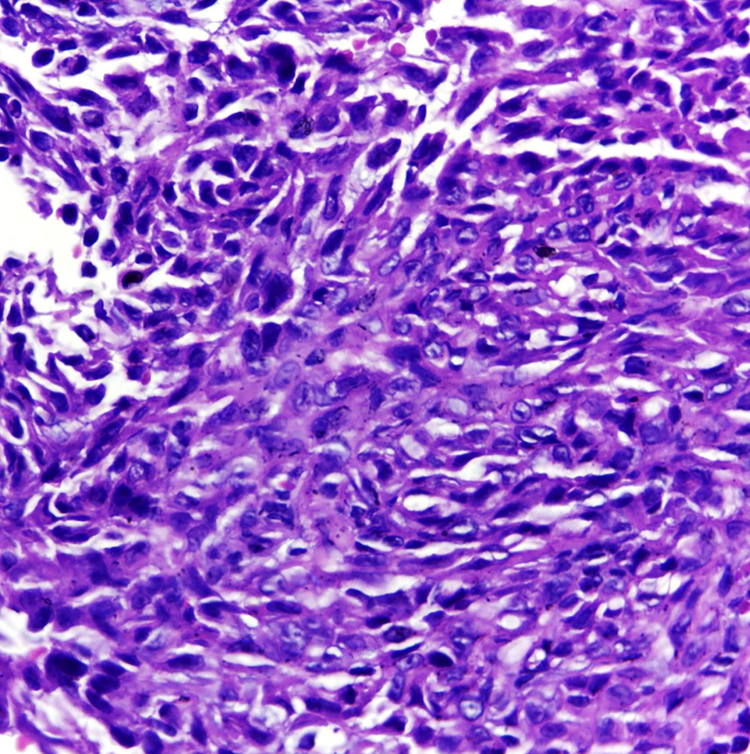
Histopathological examination showing an infiltrative neoplasm consisting of epithelioid and spindle cells with eosinophilic cytoplasm and irregular nuclei with evident nucleoli (Hematoxylin & eosin, ×400).

## Financial support

None declared.

## Authors’ contributions

Maraya de Jesus Semblano Bittencourt: Approval of the final version of the manuscript; collection, analysis, and interpretation of data; critical review of the literature; critical review of the manuscript.

Caren dos Santos Lima: Drafting and editing of the manuscript; collection, analysis, and interpretation of data; critical review of the literature; critical review of the manuscript.

Aline de Lima Days: Approval of the final version of the manuscript; Drafting and editing of the manuscript; intellectual participation in propaedeutic and/or therapeutic conduct of studied cases; critical review of the literature; critical review of the manuscript.

Camilla Côrrea Neri: Drafting and editing of the manuscript; collection, analysis, and interpretation of data; critical review of the literature; critical review of the manuscript.

## Conflicts of interest

None declared.

## References

[1] Thway K., Jones R.L., Noujaim J., Fisher C. (2016). Epithelioid sarcoma: diagnostic features and genetics. Adv Anat Pathol..

[2] Nunes L.F., Fiod N.J.J., Vasconcelos R.A.T., Meohas W., Rezende J.F.N. (2010). Epithelioid sarcoma: clinical behavior, prognostic factors and survival. Rev Col Bras Cir..

[3] Fleury L.F.F., Sanches J.A. (2006). Primary cutaneous sarcomas. An Bras Dermatol..

[4] Akpinar F., Dervis E., Demirkesen C., Akpinar A.C., Ergun S.S. (2014). Epithelioid sarcoma of the extremities. Indian J Dermatol Venereol Leprol..

[5] Burgos A.M., Chávez J.G., Sánchez J.L., Sánchez N.P. (2009). Epithelioid sarcoma: a diagnostic and surgical challenge. Dermatol Surg..

